# Histopathologic effects of mobile phone radiation exposure on the testes and sperm parameters: a systematic literature review of animal studies

**DOI:** 10.3389/frph.2024.1515166

**Published:** 2025-01-17

**Authors:** Ebrahim Msaye Assefa, Seid Mohammed Abdu

**Affiliations:** Department of Biomedical Sciences (Clinical Anatomy), School of Medicine, College of Medicine and Health Sciences, Wollo University, Dessie, Ethiopia

**Keywords:** histopathology, mobile phone radiation, testes, sperm parameters, lab animals

## Abstract

**Introduction:**

Male infertility, often attributed to insufficient production of healthy and active sperm, can be exacerbated by electromagnetic radiation emitted from mobile phones, which disrupts normal spermatogenesis and leads to a notable decline in sperm quality. The main targets of mobile phone-induced damage in the testes are Leydig cells, seminiferous tubules, and sperm cells. The aim of this systematic literature review is to identify histopathological changes in the testes due to mobile phone radiation exposure and to examine its effects on sperm parameters in experimental animals.

**Methods:**

In this systematic review, an extensive literature search was conducted across databases such as PubMed, ScienceDirect, Hinari, and Google scholar.

**Results:**

A total of 752 studies were identified for screening, and 18 studies were deemed eligible for data extraction. Studies have identified histopathological alterations in testicular tissue caused by mobile phone radiation, such as reduced seminiferous tubule diameter, tunica albuginea and germinal epithelial thickness, Leydig cell hypoplasia, and increased intertubular space. Consistent exposure to mobile phone radiation has been shown to significantly reduce sperm count, motility, and viability, while also increasing abnormal sperm morphology in male rats, mice, and rabbits.

**Conclusion:**

Animal studies indicate that electromagnetic radiation from mobile phones can negatively impact testicular tissue and sperm parameters, including sperm count, motility, viability, and morphology. As a precaution, preventive measures are recommended to minimize potential risks from mobile phone exposure, and further research is needed to fully understand its effects on human reproductive health.

## Introduction

Infertility is the inability to conceive after one year of consistent, unprotected intercourse (1). Around 35% of infertility cases are linked to male factors (2). Commonly, male infertility stems from insufficient production of healthy and active sperm (2, 3). Several risk factors linked to male infertility include genetic abnormalities, blockage of genital ducts, varicocele, erectile dysfunction, and impotence (4). Environmental factors like heat, chemicals, radiation, alcohol, and smoking also play a role in reducing fertility (5, 6).

Over the last two decades, mobile phones have become essential to daily life, with their use increasing dramatically (7). Mobile phones operate within a spectrum of frequencies ranging from 450 to 2,700 MHz, emitting electromagnetic radiation (EMR) as they function (8). The specific absorption rate (SAR) measures the amount of radiofrequency energy absorbed by tissues from mobile phones. Depending on the model, SAR values for cell phones range from 0.12 to 1.6 W/kg body weight (9). When mobile phones are kept in pockets near the scrotum, the testes can absorb the emitted electromagnetic radiation. This has raised concerns about potential health effects on male reproductive organs, particularly the testes (10, 11). Several studies have indicated that the testes, specifically the Leydig cells, are particularly sensitive to exposure to electromagnetic radiation (12).

The impact of mobile phone on male reproduction remains uncertain due to conflicting results from various studies. Yet, it is believed that the EMR emitted by mobile phones might disrupt normal sperm production, potentially decreasing sperm quality. Studies suggest that the use of mobile phone could affect semen quality, impacting sperm count, motility, viability, and serum testosterone levels, possibly playing a role in male infertility (7, 13–15). Animal studies indicate that mobile phone exposure can lead to harmful changes in the testes and negatively affect male germ cells (16–19). Some researchers have found no harmful effects from exposure to EMR emitted by mobile phones, noting no histological changes in rat testes or alterations in serum testosterone levels (20, 21).

## Histological features and functions of testes

The testes, located in the scrotum outside the abdominal cavity, require a lower temperature for proper function and sperm production. Keeping a mobile phone in your trouser pockets and using it for extended period may elevate testicular temperature, potentially causing hyperthermia and oxidative stress (22). Oxidative stress in sperm cells, resulting in impaired fertilization ability and DNA damage, is associated with decreased fertility (23).

The testes have two primary components: the seminiferous tubules, which are responsible for sperm production, and the Leydig cells, have role for male sex hormone production. Sperm production, regulated by Y chromosome genes, generally takes about 54 days in rats from the spermatogonia stage (24). Additionally, it takes approximately 12–21 days for sperm to travel from the testis to the epididymis and then to the ejaculatory duct. During this time, sperm mature in the epididymis, acquiring motility. Leydig cells produce testosterone, which plays a crucial role in regulating spermatogenesis (10, 25).

The testes, essential for sperm development and maturation, are highly susceptible to radiation, potentially leading to genetic damage (21). Sperm cellular membranes are rich in polyunsaturated fatty acids (PUFAs), making them vulnerable to oxidative damage from reactive oxygen species (ROS), which can result in lipid peroxidation. This process undermines membrane integrity and reduces sperm motility (23). Mobile phone radiation mainly impacts the Leydig cells, seminiferous tubules, and spermatozoa. This exposure lowers testosterone production, interferes with sperm production, and damages sperm DNA (26).

Histological examination of the testis reveals seminiferous tubules, which are hexagonal or rounded in shape, separated by interstitial connective tissue. Inside these tubules, different stages of spermatogenic cells are found, but only Sertoli cells and spermatogonia from the seminiferous epithelium are situated adjacent to the basement membrane. Leydig cells, characterized by large, acidophilic cytoplasm, reside in the interstitial tissue between seminiferous tubules. The germinal epithelium contains layers of spermatogenic cells, including spermatogonia, primary and secondary spermatocytes, as well as early (round) and late (elongated). Spermatozoa are free in the lumen (27, 28).

Spermatogenesis is a synchronized, intricate, and lengthy process that takes place in the germinal epithelium of the testes (29). Testes are sensitive to stressors, both internal and external, and exposure to EMR can disrupt germinal cells at various differentiation stages, potentially causing infertility (30). This systematic literature review aims to identify histopathological changes in the testes due to mobile phone radiation exposure and to examine its effects on sperm parameters in experimental animals.

## Methods

### Literature search strategy

The study was conducted in accordance with the Preferred Reporting Items for Systematic Reviews and Meta Analysis (PRISMA) 2020 guidelines. It reviewed research reports on the histopathological effects of mobile phone radiation on the testes and sperm parameters. A thorough literature search was performed using databases like PubMed, ScienceDirect, Hinari, and Google Scholar, along with manual searches of reference lists from relevant articles and university websites to identify additional studies.

The following key words and terms were utilized to identify relevant studies: “histopathology”, “testes”, “sperm parameters”, “mobile phone exposure”, “sperm count”, “sperm motility”, “sperm viability”, “sperm morphology”, “electromagnetic radiation”, “experimental animals”.

### Eligibility criteria

#### Inclusion criteria

✓ Type of studies: animal studies with English language
✓ Only studies that included a control or comparator group were selected
✓ Year of studies: 2000 to 2021 were included in the study
✓ Participants: rats (Sprague-Dawley and Wistar rat strains), Albino mice and rabbits
✓ Complete articles on the topic of interest were included

#### Exclusion criteria

✓ Incomplete and abstract only articles, and reviews are omitted from the review.
✓ Some articles with redundant ideas were also omitted by taking the most recent one.

### Study variables

✓ Radiation exposure characteristics (e.g., frequency, duration)
✓ Testicular histopathologic changes (e.g., diameter of seminiferous tubules, thickness of tunica albuginea, number of Sertoli and Leydig cells, degeneration of germinal epithelium, etc.)
✓ Sperm parameters (e.g., sperm count, motility, morphology, and viability)

### Data abstraction and analysis

All the included studies were examined in detail and a reported result from each animal studies such as histopathological changes and sperm parameters of animals of exposed and control group were abstracted. Then the data was analyzed using qualitative analysis method and finally presented in text and tables.

## Results and discussion

**Figure 1:**
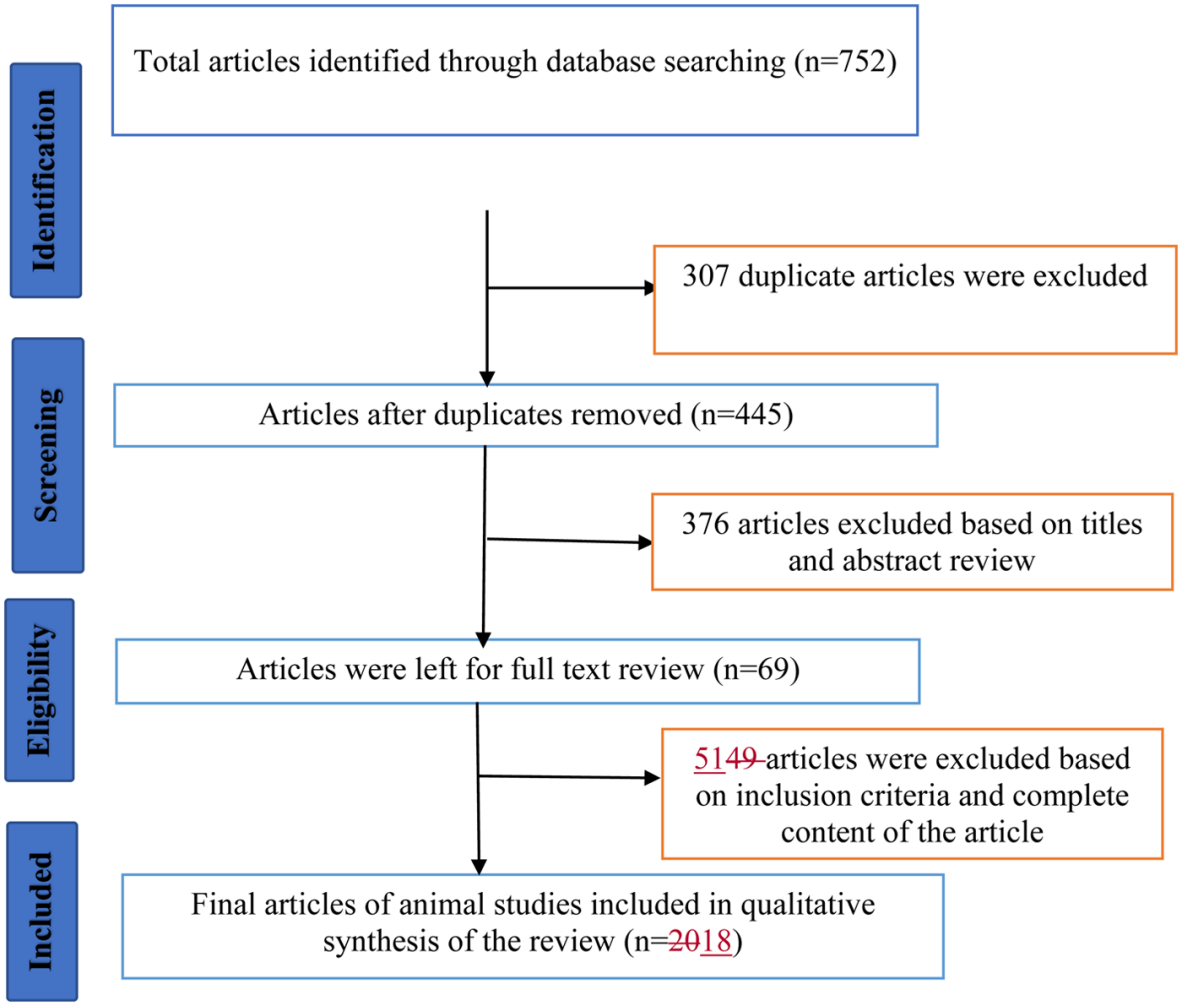
A flow diagram illustrating the review process.

A total of 752 articles were identified through keyword searches. Three hundred seven duplicate articles and an additional 376 after screening of titles and abstract, and 51 articles after screening the full of content of the studies were excluded based on the selection criteria. Finally, a total of 18 articles which fulfilled the inclusion criteria have been reviewed (Figure 1).

### Histopathological changes of the testes due to mobile phone radiation exposure

Research has explored the tissue changes in the testes resulting from mobile phone EMR exposure. These alterations are influenced by factors such as exposure length, the specific absorption rate (SAR), and the energy levels of the EMR (Table 1).

**Table 1:** Characteristics of studies included in the review that shows histopathological changes of mobile phone radiation exposure on the testes.

Authors, year	Country (Study area)	Subjects/species	Type of devices used to generate the EMR	Exposure parameters/conditions	Major histopathological changes of testes
Odaci et al. (17)	Turkey	Male Sprague- Dawley rats, *n* = 20	Radiation simulator	900 MHz; 1 h/d for 13–21 days, SAR = 0.024 W/kg	Decreased diameter of seminiferous tubule, immature germ cells were observed in the lumen of the seminiferous tubules.
Tas et al. (18)	Turkey	Male Wistar Albino rats, *n* = 14	Radiation simulator	900 MHz; 3 h/d for 1 year, SAR = 0.0623 W/kg	Decreased tunica albuginea thickness in exposed group
Mugunthan et al. (16)	India	Male Swiss Albino mice, *n* = 36	Mobile phone	900–1,800 MHz from 2G cell phone and 1,900–2,200 MHz from 3G cell phone. 48 min per day for 30 to 180 days SAR = 1.69 W/kg	The exposed group exhibited a reduced number of Sertoli and Leydig cells, detachment of Sertoli cells and spermatogonia from the basal lamina and vacuolar degeneration and desquamation of seminiferous epithelium
Singh et al. (19)	India	Male Swiss Albino mice, *n* = 36	Mobile phone	2,400 MHz; 4 h/d for 120 days, SAR = 0.402 to 1.52 W/kg	Hypo-spermatogenesis, Leydig cell hypoplasia and increased intertubular space
Farjanikish et al. (31)	Iran	Male Swiss Albino mice, *n* = 30	Mobile phone	The cell phones were kept in standby mode all day and in talking mode for 150 min daily over a period of three months	Seminiferous tubules cellular necrosis, an infiltration of inflammatory cells into the parenchymal tissue, a reduction in the Leydig cell count, multinucleated cells (syncytium formation)
Nisbet et al. (32)	Turkey	Male Wistar Albino rats, *n* = 33	Radiation simulator	900 to 1,800 MHz; 2 h/d for 90 days, SAR = 0.011 to 3 W/kg	Severe vacuolar degeneration of the germinal epithelium was observed, characterized by pyknotic cells, extensive necrosis, and desquamation of the epithelium
Nassar et al. (33)	Egypt	Pregnant Albino mice and their pups, *n* = 10	Mobile phone	800 to 1,800 MHz; 0.5 h/d for 45 days, SAR = 0.85 to 1.06 W/kg	Apoptotic spermatogonia, injured spermatocytes and spermatids, vacuolar degeneration within the tubules, and the degeneration of Leydig cells
Farag and Yousry (34)	Egypt	Male Albino rats, *n* = 24	Mobile phone	900 MHz; 1 h/d for 8 weeks	There was a widening of interstitial spaces, accompanied by irregular basement membranes in some seminiferous tubules and detached spermatogenic cells that resulted in empty spaces and multinucleated giant cells
Hasan et al. (28)	Bangladesh	Male Swiss Albino mice, *n* = 30	Mobile phone	2,400 MHz; 40 min and 60 min per day for 2 months, SAR = 0.087 W/kg	Irregular shapes and non-uniform sizes, as well as a reduced layer of spermatogenic cells, contributed to the enlarged lumen of the seminiferous tubules
Hegazy et al. (35)	Egypt	Pregnant Albino rats, *n* = 16	Mobile phone	900–1,800 MHz; 2 h/d starting from the sixth day of pregnancy and continuing through lactation until the weaning period, SAR = 2.0–1.23 W/kg	Distorted seminiferous tubules with destructed germinal epithelium and germinal cells were observed exfoliating into the lumen. Homogenous acidophilic material deposition also appears between the interstitial cells. Some Sertoli cells in the exposed rats exhibited vacuoles in their cytoplasm.
Kumar and Shukla (36)	India	Male Swiss Albino rats, *n* = 24	Mobile phone	Code Division Multiple Access (CDMA) mobile phone; 3 h day for five months	Vacuolated irregular shaped mitochondria, pyknotic nuclei in germ cells, and numerous lipid droplets were observed in the cytoplasm
Lee et al. (21)	Korea	Male Sprague-Dawley rats, *n* = 40	Radiation simulator	848.5 MHz; 90 min/day, 5 days/week, for 12 weeks, SAR = 2.0 W/kg	No effect on seminiferous tubule diameter and no histopathological alterations in the testis, such as cell debris, pyknotic nuclei, or seminiferous tubule atrophy in the exposed group
Kim et al. (20)	Korea	Male Sprague-Dawley rats, *n* = 20	Radiation simulator	2.45 GHz EMF for 1 h or 2 h a day. SAR = 1.41 W/kg	An increase in the number of Leydig cells was observed in the exposed group

Exposure to extremely low-frequency irradiation may result in decreased sperm count and compromised reproductive function in the testes (37). Electromagnetic radiation can enhance oxidative stress by disrupting the balance between the production of reactive oxygen species (ROS) and the body's antioxidant defense mechanisms can lead to cellular damage (38). Due to high metabolic rates and cell replication in the testes, oxidative stress presents a considerable threat, especially in the seminiferous tubules. Exposure to EMR and heat can weaken the blood-testis barrier, resulting in degeneration of spermatogonia (39).

Extended exposure to 2.4 GHz EMR induces changes in the overall morphology of rat testes, resulting in a decrease in the diameter of seminiferous tubules was observed in the exposed rats (40). EMR exposure decreased both the diameter of the seminiferous tubules and the thickness of the germinal epithelium (17, 41). Saygin et al. indicated that short-term exposure to 2.45 GHz EMR did not result in any changes to the diameter of the seminiferous tubules (42). The difference in exposure duration between the studies could account for this variation. In addition, Lee et al. also found that exposure to 848.5 MHz RF for 12 weeks had no effect on seminiferous tubule diameter (21).

Tas et al. found that a reduction in tunica albuginea thickness in rats subjected to prolonged exposure to 900 MHz radiofrequency radiation from mobile phones (18). Dasdag et al. similarly found that a reduction in tunica albuginea thickness was observed in the exposed rats (40). The tunica albuginea's contractile properties help propel spermatozoa from the testes into the epididymis (43). Decreased production of Type I, Type III, and Type V collagens, as well as decreased fibroblast proliferation capacity, could lead to the thinning of the tunica albuginea (44).

Prolonged exposure to 2G cell phone radiation in mice caused a marked decrease in testis weight and seminiferous tubule diameters. Additionally, exposure to 2G radiation in mice led to a significant reduction in the number of Sertoli and Leydig cells, resulting in a significant drop in serum testosterone levels (45). Additional studies have indicated that cell phone EMR can cause Leydig cell hypoplasia and an increase in intertubular space (19, 31, 36, 46–48). Leydig cells are particularly vulnerable to EMR, which may adversely affect their structure and function, leading to reduced serum testosterone levels (49). In contrast, research by Kim et al. indicated that prolonged exposure of rats to 2.45 GHz radiation resulted in a rise in the number of Leydig cells and higher serum testosterone levels (20). Another study also indicated that the mean number of Sertoli cells in the seminiferous tubule showed no significant difference between the exposed and control groups (21).

Exposure to EMR negatively impacts the structure of the germinal epithelium within seminiferous tubules. Spermatogenic cells in the epithelium were observed to be widely separated by empty spaces, with nuclei condensation evident in dividing cells. The exposed group displayed numerous atypical tubules at various stages of sperm development, characterized by disorganized or absent germinal epithelium. Exposing the testes to 1800 MHz EMR resulted in abnormal cellular morphology in rats, characterized by severe vacuolar degeneration of the germinal epithelium was observed, along with the presence of pyknotic cells and significant necrosis and desquamation of the epithelium (32). A related study similarly detected vacuolation and giant cells in the germinal epithelium, along with the presence of abnormal cells in the lumen of the seminiferous tubules within the exposed group (50).

Nassar et al. examined the impacts of exposure to non-ionizing radiation from mobile phones on mice pups during both the intrauterine period and after birth. They observed various histopathological abnormalities in the testes, including disorganization and damage to germ cells located in the seminiferous tubules. The study further showed the occurrence of apoptotic spermatogonia, compromised spermatocytes and spermatids, intra-tubular vacuolar degeneration, and the degeneration of Leydig cells (33). Naggar et al. found that exposed male rats to 950 MHz EMR for three hours each day over a period of two months led to degeneration, disorganization, and atrophy in certain seminiferous tubules, along with expanded interstitial spaces. They also observed ruptured basement membranes with detached cells, reduced cellularity, and significantly diminished sperm count within the seminiferous tubule lumens (48). In contrast, another study conducted by Lee et al. exposed male rats to 848.5 MHz CDMA cellular phone phone-based RF of for 12weeks, and found no histopathological changes, including cell debris, pyknotic nuclei, or seminiferous tubule atrophy, in the exposed group (21).

Exposing mice to mobile phones in standby mode all day and in talking mode for 150 min each day over 90 days led to histopathological alterations in their testes. The study revealed cellular necrosis in seminiferous tubules and infiltration of inflammatory cells into the parenchymal tissue. Additionally, some necrotized tubules displayed the presence of multinucleated cells, indicating syncytium formation (31). Another study conducted in male rats exhibited irregular seminiferous tubules, a reduced number of spermatogonia, and the presence of giant multinucleated cells, and sparse primary spermatocytes with densely condensed nuclei. Additionally, degenerated spermatocytes were observed within the lumens of seminiferous tubules in rats exposed to EMR (10).

Farag and Yusry's study illustrated distorted histological architecture in certain seminiferous tubules, characterized by widened interstitial spaces. Additionally, irregular basement membranes were observed, along with separated spermatogenic cells, leaving behind empty spaces. Furthermore, giant cells with multiple nuclei were noted (34). The widening of interstitial spaces may result from impaired spermatogenesis, leading to a reduction in the height of the germinal epithelium alongside the diameter of the seminiferous tubules. Multinucleated giant cells could arise from the opening of cytoplasmic bridges develop between progeny cells, resulting in the merging of their cellular contents (51).

Histopathological analysis of mouse testes exposed to 60 min of EMR from a 4G cell phone revealed irregularly shaped and non-uniformly sized seminiferous tubules. These tubules exhibited fewer layers of spermatogenic cells, resulting in larger lumens devoid of spermatozoa (28). Esa et al. also found that EMR induced notable alterations within the seminiferous tubules of the mice that were exposed, leading to irregular shapes and dimensions (52). Chauhan et al. found alterations in the epithelial lining of seminiferous tubules, along with a reduction in the size of the tubule lumens, along with reduced cell population, in mice exposed to radiation (53).

Hegazy et al. explored the impacts of cell phone radiation exposure occurring both before and after birth on rat testicular development, uncovering degenerative alterations. These changes included distorted seminiferous tubules with disrupted germinal epithelium and the shedding of germinal cells into the lumen. Additionally, deposition of homogeneous acidophilic material was noted among the interstitial cells (35). Exfoliated germinal cells may result from disrupted connections between Sertoli cells and developing germ cells (51). The appearance of homogeneous acidophilic material in some tubules may result from reduced phagocytic activity of Sertoli cells (34).

Exposure to a mobile phone for five months induces ultrastructural alterations in rat testicular tissue. Findings include irregularly shaped, vacuolated mitochondria, pyknotic nuclei in germ cells, and an abundance of lipid droplets in the cytoplasm (36). Pyknotic nuclei observed in germ cells could be attributed to the degeneration of germ cells at various developmental stages following radiation exposure. Additionally, another study noted the presence of vacuoles in the cytoplasm of certain Sertoli cells in exposed rats (35). Vacuoles seen in Sertoli cells may be due to the buildup of lipid droplets that nourish germ cells (54).

### Effect of mobile phone radiation exposure on sperm parameters

Research on experimental animals such as rats and mice has demonstrated that exposure to mobile phone radiation negatively impacts sperm parameters, including a significant reduction in epididymal sperm count, motility, viability, and an increase in abnormal sperm morphology (Table 2).

**Table 2:** Overview of the studies included in the review that demonstrate the effects of mobile phone radiation exposure on sperm parameters.

Authors, year	Country (study area)	Subjects/species	Type of devices used to generate the EMR	Exposure parameters/conditions	Sperm parameters
Lee et al. (21)	Korea	Male Sprague-Dawley rats, *n* = 40	Radiation simulator	848.5 MHz; 90 min/day, 5 days/week, for 12 weeks, SAR = 2.0 W/kg	No significant alterations in sperm counts
Odaci et al. (17)	Turkey	Male Sprague- Dawley rats, *n* = 20	Radiation simulator	900 MHz; 1 h/d for 13–21 days, SAR = 0.024 W/kg	No significant difference in total sperm counts was found between the exposed group and the control group, but a significant decrease in motility and viability was noted
Adebayo et al. (55)	Nigeria	Male Albino rats, *n* = 30	Telecommunication network	1,800 MHz; 1 h/d for 5weeks, SAR = 1.4 W/kg	Decreased sperm count and motility
Oh et al. (56)	Korea	Male Sprague-Dawley rats, *n* = 20	Radiation simulator	2.104 GHz; 6 h/d for 28 days, SAR = 3.0msW/kg	Sperm or germ cells count and motility significant decreased in the exposed rats
Meo et al. (57)	Saudi Arabia	Male Wistar Albino rats, *n* = 40	Mobile phone	900 MHz; 30 min–60 min/d for 3 months, SAR = 0.053 W/kg	Reduced sperm production and arrested maturation of spermatozoa in the testes of rats
Oyewopo, et al. (58)	Nigeria	Male Wistar Albino rats, *n* = 20	Mobile phone	900/1,800 MHz; 1 h–3 h/day for 28 days, SAR = 1.23 W/kg	Exposed rats exhibited a significant reduction in sperm viability, along with a notable decline in normal sperm morphology
Banavath and Srinivasa (59)	India	Male Sprague Dawley rats, *n* = 30	Mobile phone	800 MHz to 2 400 MHz; 60 min/day for 90 days, SAR = 0.53 W/kg	In the exposed rats, sperm count, viability, and motility were significantly reduced, while abnormalities in sperm morphology—including detached heads, pyriform heads, coiled tails, and bent tails—were significantly increased
Singh et al. (19)	India	Male Swiss Albino mice, *n* = 36	Mobile phone	2,400 MHz; 4 h/d for 120 days, SAR = 0.402 to 1.52 W/kg	Decline in sperm count, sperm motility, and serum testosterone levels in the exposed group
Nisbet et al. (32)	Turkey	Male Wistar Albino rats, *n* = 33	Radiation simulator	900 to 1,800 MHz; 2 h/d for 90 days, SAR = 0.011 to 3 W/kg	Enhanced forward motility of epididymal sperm and normal sperm morphology in rats and serum testosterone level
Farag and Yousry (34)	Egypt	Male Albino rats, *n* = 24	Mobile phone	900 MHz; 1 h/d for 8 weeks	Significantly decreased sperm count
Hegazy et al. (35)	Egypt	Pregnant Albino rats, *n* = 16	Mobile phone	900–800 MHz; 2 h/d starting from the sixth day of pregnancy and continuing through lactation until the weaning period, SAR = 2.0–1.23 W/kg	Significantly decreased sperm count and motility
Kim et al. (20)	Korea	Male Sprague-Dawley rats, *n* = 20	Radiation simulator	2.45 GHz EMF for 1 h or 2 h a day. SAR = 1.41 W/kg	The number of spermatocytes was significantly decreased, and no sperms with abnormal morphology were observed

In rats, sperm is primarily collected through epididymal retrieval. Due to their small body size, the testes can readily and regularly move between the abdomen and scrotum through the inguinal canal (60). Mobile radiation exposure in male rats caused a significant reduction in both epididymal sperm count and motility (61). Another study revealed that exposure to mobile phones for 6 h daily over 5 days leading to a reduction in the rapid progressive motility of sperm cells (14). Odaci et al. examined the effects of prenatal exposure to 900 MHz EMR on rat testicular health and epididymal sperm quality, finding reduced sperm motility and immature germ cells in the seminiferous tubule lumen of exposed rats (17).

Several studies also reported decreased sperm count and motility in rats and mice exposed to non-ionizing radiation (27, 55, 62–65). Exposure to EMR adversely impacts sperm parameters, causing significant alterations in the sperm cell cycle due to impaired Leydig and Sertoli cells that are crucial for cell proliferation (66). EMR exposure alters cellular enzyme activity, leading to reduced production of adenosine triphosphate (ATP), essential for sperm motility. Experimental research indicates that mobile phone radiation elevates oxygen free radical levels, leading to oxidative stress (67, 68). Oxidative stress in testicular tissue impairs Leydig cell function (69) and disrupts the ability of the germinal epithelium to produce normal sperm cells or undergo spermatogenesis (70). The localized thermal effects of EMR may contribute to a decrease in sperm count (71).

Long-term exposure to mobile phone radiation may harm spermatogenesis. The study found a marked decrease in sperm cell count, along with reduced motility in rats exposed to radiation (56). This could be due to a reduction in Leydig cell count, responsible for secreting testosterone, which in turn promotes spermatogenesis. Long-term exposure to mobile phone radiation caused hypospermatogenesis and arrested the maturation of spermatozoa in the testes of Wistar albino rats (57). This could be attributed to hormonal disruption induced by EMR. Meo et al. examined the effects of mobile phone radiation on serum testosterone levels in Wistar albino rats, revealing a significant reduction in testosterone levels among those exposed to radiation (72). Mobile phone radiation impacts reproductive morphology and alters hormone levels, such as serum testosterone and FSH, which are vital for spermatogenesis and the maturation of spermatozoa (73, 74). Conversely, another study reported no significant alterations in sperm counts including the number of spermatocytes and round spermatids, during spermatogenesis in rats exposed to mobile phone EMR (21).

Several studies have also observed a marked reduction in sperm viability among exposed rats (58, 59, 75–78). EMR could impact cell function by altering ion channel structure and plasma membrane integrity. The decrease in sperm viability may be attributed to EMF-induced disruptions in sperm cell membrane mechanisms regulating ion flow, particularly sodium and potassium, ultimately affecting water content and sperm viability (79).

Hamdi et al. explored the impact of EMR exposure during the developmental stages of mice on testicular tissues in adulthood. The research identified several sperm abnormalities, including double-tailed sperms, sperms without tails, sperm exhibiting abnormal head shapes, and those with cytoplasmic droplets (27). Another study indicated a notable rise in aberrations in sperm morphology, including detached heads, pyriform heads, coiled tails, and bent tails, among rats exposed to EMR (59). Studies have reported DNA damage in sperm cells following EMR exposure to the testes. This damage may correlate with the abnormalities seen in the sperm head and the mitochondrial sheath of the sperm tail (46, 80). However, a study by Kim et al. on the long-term exposure of rats to a 2.45 GHz electromagnetic field found no significant effects on sperm morphology (20). Defective sperm function is a major cause of male infertility. Excessive exposure of sperm to mobile phone radiation disrupts sperm function by affecting the mitochondria located in the mid-piece of the sperm tail, which are crucial for ATP production. This disruption leads to the generation of high levels of reactive oxygen species (ROS), contributing to decreased sperm motility. Studies have shown alterations in microtubule arrangement following exposure to mobile phone radiation. In experiments with EMR-exposed rats, significant changes in microtubules were observed, resulting in abnormalities in the sperm tail and adversely affecting motility. Additionally, radiation can disrupt the acrosome, potentially impairing spermatozoa's ability to penetrate oocytes, thus contributing to infertility (23, 81).

### Limitations

The included studies varied significantly in terms of experimental design, radiation exposure protocols, animal models, and histopathologic assessment methods. This heterogeneity limited the ability to draw consistent conclusions and precluded a meta-analysis. The studies used parametric tests without verifying the assumptions like normality and homogeneity of variances, which might introduce biases and affect the reliability of the findings. The studies also had small sample sizes, which may affect the statistical power and generalizability of the results.

## Conclusion

Animal studies indicate that electromagnetic radiation from mobile phones can negatively impact testicular tissue histology and various sperm parameters, potentially affecting sperm count, motility, viability, and morphology. Therefore, it is advisable to exercise caution and implement preventive measures to reduce the potential risks associated with mobile phone use. Additionally, further research is essential to gain a comprehensive understanding of the effects of mobile phone radiation on human reproductive health.

## Data Availability

The original contributions presented in the study are included in the article/Supplementary Material, further inquiries can be directed to the corresponding author.
